# The Impact of Warming on Assembly Processes and Diversity Patterns of Bacterial Communities in Mesocosms

**DOI:** 10.3390/microorganisms11112807

**Published:** 2023-11-19

**Authors:** Qian Yang, Yifeng Yan, Jinhe Huang, Zhaolei Wang, Mingjun Feng, Haowu Cheng, Peiyu Zhang, Huan Zhang, Jun Xu, Min Zhang

**Affiliations:** 1 Hubei Provincial Engineering Laboratory for Pond Aquaculture, Engineering Research Center of Green Development for Conventional Aquatic Biological Industry in the Yangtze River Economic Belt, College of Fisheries, Huazhong Agricultural University, Wuhan 430070, China; yang123321@foxmial.com (Q.Y.); yanyifeng2@163.com (Y.Y.); huangjinhe@webmail.hzau.edu.cn (J.H.); w13483003139@163.com (Z.W.); 17671718581@163.com (M.F.); chenghw3829@163.com (H.C.); 2 Institute of Hydrobiology, Chinese Academy of Sciences, Wuhan 430072, China; zhangpeiyu@ihb.ac.cn (P.Z.); zhanghuan@ihb.ac.cn (H.Z.); xujun@ihb.ac.cn (J.X.)

**Keywords:** climate warming, bacterioplankton, sedimentary bacteria, multi-seasons, community composition, assembly process

## Abstract

Bacteria in lake water bodies and sediments play crucial roles in various biogeochemical processes. In this study, we conducted a comprehensive analysis of bacterioplankton and sedimentary bacteria community composition and assembly processes across multiple seasons in 18 outdoor mesocosms exposed to three temperature scenarios. Our findings reveal that warming and seasonal changes play a vital role in shaping microbial diversity, species interactions, and community assembly disparities in water and sediment ecosystems. We observed that the bacterioplankton networks were more fragile, potentially making them susceptible to disturbances, whereas sedimentary bacteria exhibited increased stability. Constant warming and heatwaves had contrasting effects: heatwaves increased stability in both planktonic and sedimentary bacteria communities, but planktonic bacterial networks became more fragile under constant warming. Regarding bacterial assembly, stochastic processes primarily influenced the composition of planktonic and sedimentary bacteria. Constant warming intensified the stochasticity of bacterioplankton year-round, while heatwaves caused a slight shift from stochastic to deterministic in spring and autumn. In contrast, sedimentary bacteria assembly is mainly dominated by drift and remained unaffected by warming. Our study enhances our understanding of how bacterioplankton and sedimentary bacteria communities respond to global warming across multiple seasons, shedding light on the complex dynamics of microbial ecosystems in lakes.

## 1. Introduction

With continued warming this century, extreme weather events, such as heatwaves and large temperature fluctuations, are projected to become more frequent [1,2]. Ongoing climate warming is threatening global biodiversity and altering ecosystem functions, which, in turn, can lead to feedback on climate warming [3]. Lakes are generally considered to be more vulnerable to global warming than air and oceans [4,5].

Aquatic microbial communities are highly dynamic and continually change in response to seasonal environmental variables (e.g., temperature and nutrients) [6,7]. The composition, activity, and assembly mechanisms of microbial communities have been shown to change seasonally in freshwater environments. Aquatic microbial communities are highly diverse, and the extent to which this diversity arises from deterministic factors, such as ecological niche differentiation, or stochastic factors, such as ecological drift, is debated [8,9]. Deterministic processes affect the fitness of microbial communities and determine their composition and abundance [10], whereas stochastic processes lead to unpredictable community changes [11]. The dominance of deterministic processes results in a community that is well adapted to environmental conditions, whereas the dominance of stochastic processes increases the likelihood of a high proportion of maladapted taxa and may reduce some biogeochemical functions at the microbiome level [12]. The cumulative balance of deterministic and stochastic processes affects microbiome composition, which in turn affects ecosystem function [13]. Assembly theory provides a framework to better understand microbiome changes and their impact on community function and climate feedback, helping to disentangle the mechanisms of microbial communities in response to climate change [14,15].

The water column and sediment are two distinct lacustrine habitats [16], which interact in complex patterns through biogeochemical processes in lake systems [17,18,19]. There is a distinct difference in the composition of the community structure between the water and sediment [20,21]. However, to fully understand the ecological and biogeochemical implications of climate change, we need to consider climatic influences on community structure and microbiome assembly mechanisms across aquatic habitats. Physicochemical and biological constituents in the water column can settle into the surface sediments and can be resuspended from the surface sediments back into the water column [22,23]. The abiotic and biotic properties of the water column and surface sediments are inherently intertwined and inseparable [24]. Bacteria are key components of biogeochemical cycles and food webs in various aquatic ecosystems [25,26] and are commonly found in the water column and sediments, maintaining the stability of ecological functions and services [27]. Many planktic species form dormant or resting stages (propagules) that accumulate in aquatic sediments over time and remain viable for centuries [28,29]. Elucidating their spatio-temporal variation and potential drivers is widely regarded as a fundamental ecological goal, with important implications for predicting ecosystem responses to environmental change and regulating the ecosystem function [30].

How climate warming will affect microbial communities is a matter of debate [31,32,33,34]. Temperature has important effects on microbial growth and physiology, and previous studies have shown that microbes are sensitive to changes in environmental temperature [35,36]. Different microbial groups have optimal temperature ranges for growth and activity [37]. While individual microbes shift their physiology in response to temperature changes, the dominance of a population within a community will change based on competition between groups with different growth rates at different temperatures or environmental conditions, thus affecting the composition of the microbial community [38]. Temperature also mediates the balance between stochastic and deterministic assembly processes in bacteria. One study found that temperature and ferrous iron were the main factors mediating the balance between stochastic and deterministic assembly processes in sediment communities [39], while another found that warming and nutrient enrichment did so [40]. Nevertheless, little attention has been paid to investigating the underlying mechanisms of bacteria in water and sediment in response to global warming and rapidly changing weather events through experimental manipulation in mesocosms. The generality of bacterial assembly patterns across lake water and sediment remains uncertain, limiting our ability to predict biogeochemistry and microbial community responses to climate change.

Based on the above findings, we aimed to investigate (1) how planktonic and sedimentary bacterial communities respond to different warming scenarios, (2) what the seasonal differences in the response of bacterial communities to warming, and (3) which ecological processes mediate the bacterial assembly in water and sediment. We tested the community structure, co-occurrence patterns, and assembly mechanisms of the sedimentary and planktonic bacterial communities in the 18 mesocosms and showed the temporal changes in the community. The effects of stochastic and deterministic processes on the bacterial community were elucidated, and their drivers were identified. At the same time, co-occurrence networks were used to explore how ecological processes affect putative interactions between taxa. The results may provide helpful information for better understanding and predicting the response of lacustrine lakes in a warming future.

## 2. Materials and Methods

### 2.1. Mesocosm Experiment Setup

The mesocosms used in our experiment are situated at the Huazhong Agricultural University in Wuhan City, Central China (30°29′ N; 114°22′ E). The experimental design of this study has been described previously [41,42] and will only be briefly described here. The experimental setup included 18 fully mixed outdoor mesocosms (diameter = 1.5 m, height = 1.4 m, and lake sediment = 0.1 m) to mimic shallow lake ecosystems. Heating was provided by a heater (Xin Shao Guang A10-3, Xin Shao Guang Technology Co., Ltd., Wuhan, China) and temperature sensors (Maxim IC DS18B20, Maxim Integrated Products, Inc., San Jose, CA, USA) connected to each tank, allowing us to monitor the temperature in each heated and unheated treatment pair in real time [42]. Each tank had a 10 cm deep, well-mixed bed layer of lake sediment and was filled with 20 μm mesh filtered lake water collected from Liangzi Lake at a depth of 1 m. Prior to the start of the experiment, all tanks were allowed to colonize under ambient conditions for two months (November to December 2018) to simulate the conditions of natural ecosystems as closely as possible and to reflect natural water bodies.

We conducted a long-term and multi-seasonal study using 18 outdoor mesocosms with the aim of investigating the microbial communities’ long-term adaptation and evolutionary processes in water and sediments. These mesocosms simulate shallow lake systems under three temperature scenarios with six replicates: (1) control (‘C’, environment temperature); (2) constant warming (‘T’, +4 °C above control temperature) according to the IPCC climate scenario RCP8.5 [43]; and (3) heatwave (‘H’, with water temperatures fluctuating ±4 °C relative to constant warming).

### 2.2. Samples Collection and Environmental Parameters

Water samples were collected from each tank every 2 weeks in winter and every week in the other seasons using a Plexiglas tube (diameter 70 mm; length 1 m). Total nitrogen (TN) and total phosphorus (TP) were determined by spectrophotometry (Unico UV-2800, Unico Scientific Instruments, Shanghai, China) after digestion with alkaline potassium persulfate [34]. Water was filtered through GF/C filters in order to determine PO_4_^3−^-P concentration using the molybdenum blue method [44], NH_4_^+^-N concentration in filtered water was determined using Nessler’s reagent colorimetric method [44], and NO_3_^−^-N was determined using spectrophotometric microdetermination method employing chromotropic acid reagent [45,46]. Chlorophyll-a was determined by water filtration through Whatman GF/C filters and spectrophotometric analysis (Unico UV-2800, Unico Scientific Instruments, Shanghai, China ) after ethanol extraction [47]. Dissolved oxygen (DO), pH, and conductivity were measured using HACH HQD Portable Meters (HACH HQ40d, Hach, Loveland, CO, USA).

Sediment samples were simultaneously collected alongside water samples and subjected to rigorous analysis using established methodologies. Upon collection, fresh samples underwent meticulous thawing, homogenization, and subsequent processing to quantify critical parameters, including TN, NH_4_^+^-N, NO_3_^−^-N, NO_2_^−^-N, TP, and inorganic phosphorus (IP) [48]. The determination of TN and TP was accomplished through the application of the peroxysulfate-assisted digestion method (HJ 832-2017). The quantification of NH_4_^+^-N, NO_3_^−^-N, and NO_2_^−^-N levels was achieved utilizing precise spectrophotometric techniques in accordance with the potassium chloride processing protocol (HJ 634-2012). Furthermore, the determination of IP was conducted using the sodium hydrogen carbonate solution–Mo–Sb anti spectrophotometric method (HJ 704-2014), ensuring the robustness and accuracy of the analytical procedures.

Bacterioplankton and sediment microbial samples were collected quarterly throughout the year (i.e., May, August, and November of 2018 and February of 2019). To obtain these samples, 100 mL of water from each tank was filtered through a 0.22 μm white polycarbonate membrane. The filtered water was subsequently preserved at −80 °C until DNA extraction. Likewise, the sediment microbial samples were also stored at −80 °C until DNA extraction.

### 2.3. DNA Extraction, PCR, and Sequencing

Water and sediment samples for molecular analysis were stored in a freezer at −80 °C prior to use. Genomic DNA from water and sediment samples was extracted and purified using the MOBIO PowerWater^®^ DNA Isolation Kit (MO BIO Laboratories, Carlsbad, CA, USA) according to the manufacturer’s protocols. The barcoded primer sets 338F (5’-ACTCCTACGGGAGGCAGCAG-3’) and 806R (5’-GGACTACHVGGGTWTCTAAT-3’) [44] were used for bacterial 16S rRNA gene amplification. The V4–V5 region of the bacterial 16S ribosomal RNA gene was amplified by PCR (95 °C for 2 min, followed by 25 cycles of 95 °C for 30 s, 55 °C for 30 s, and 72 °C for 30 s, and a final extension at 72 °C for 5 min). PCR reactions were performed in a 20 μL reaction system containing 4 μL of FastPfu buffer (5×), 2 μL of dNTP mix (2.5 mM), 0.8 μL of each primer (5 μM), 0.4 μL of FastPfu polymerase, and 10 ng of template DNA, and 0.2 μL of BSA. Triplicate amplifications from each sample were mixed for library preparation.

Amplicons were extracted from 2% agarose gels and purified using the AxyPrep DNA Gel Extraction Kit (Axygen Biosciences, Union City, CA, USA) according to the manufacturer’s instructions and quantified using QuantiFluor™-ST (Promega, Madison, WI, USA). Purified amplicons were pooled in equimolar amounts and sent for paired-end sequencing on an Illumina Miseq PE300 platform (Majorbio, Shanghai, China).

Raw reads were trimmed using Trimmomatic (version 0.38) and Flash (version 1.2.11) to remove those of low quality (<20) and short length (<50 bp). Briefly, the raw reads were combined, denoised, trimmed, quality-filtered, and aligned [49] to the SILVA 128 databases using Mothur (version 1.30.2). After initial processing, UCHIME was used to remove chimera, and operational taxonomic units (OTUs) were clustered at a 97% sequence similarity level [50] using Usearch (version 7.0). To avoid potential sequencing bias, all singletons and OTUs occurring in only one sample were excluded from the OTU table.

### 2.4. Network Construction and Analysis

The total datasets were divided into two subgroups: water and sediment samples, which were analyzed individually. Network analysis was performed at the microbiome OTU level, and OTUs with total relative abundance > 0.1% were selected to reduce computational complexity and ensure the reliability of the constructed network [51]. Only Spearman’s rank correlation coefficients > 0.6 (or <−0.6) and *p* < 0.05 were accepted for network analysis [52,53]. Corrections for multiple testing were considered, and *p*-values were corrected for a false discovery rate (FDR) of 0.05 using the Benjamini–Hochberg method [54]. The resulting correlations were imported into the Gephi platform (version 0.10.1) [55] and then visualized using the Fruchterman–Reingold algorithm. The microbial co-occurrence network’s properties were characterized using several properties, including nodes, edges, average degree, average transitivity, average clustering coefficient, average path length, diameter, modularity, graph density, and percentage of positive relationships. These properties collectively provide insight into the network structure, connectivity, organization, and prevalence of cooperative interactions among microbial community members. The topological properties of the networks were also computed in Gephi.

### 2.5. Bacterial Community Assembly Analysis

Interspecific phylogenetic distances were calculated using the maximum likelihood method in MEGA 7 [56,57]. To investigate the mechanisms of bacterial community assembly, the mean nearest taxon distance (MNTD) and the standardized effect size (SES) of the MNTD (opposite number of NTI) were calculated using the ‘ses.mntd’ function in the ‘picante’ package of R [58,59,60]. Overall, if the NTI values are between −2 and 2, the community assembly for a given pairwise comparison is considered to be primarily driven by dispersal-related or stochastic processes. However, if the NTI values are outside of this range (−2 to 2), then deterministic processes have a more important function in structuring the turnover of the microbial phylogeny [61]. To explore the contribution of five ecological processes (including homogenizing selection, variable selection, dispersal limitation, homogenizing dispersal, and ecological drift) to bacterial community structure, we applied a quantitative ecological framework based on null models, as previously reported [62]. First, βNTI was calculated. Values >  +2 or <−2 indicate that deterministic processes influence community structure, whereas the values between −2 and  +2 indicate that stochastic processes are highly influential [63]. Second, βNTI and Bray–Curtis-based Raup–Crick (RCbray) were combined and used to infer the relative importance of key ecological processes in influencing bacterial communities [63]. βNTI values >  +2 or <−2 indicate that community turnover is determined by variable or homogeneous selection (similar community composition under consistent environmental conditions), respectively. Pairwise comparisons with RCbray > +0.95 and |βNTI| < 2 correspond to dispersal limitation (high compositional turnover is mainly caused by low dispersal rates, allowing community composition to drift apart). RCbray < −0.95 and |βNTI| < 2 correspond to homogenizing dispersal, which is similar to mass effects and source-sink dynamics; however, these terms invoke additional assumptions and processes. Therefore, homogenizing dispersal simply indicates that dispersal is high enough to cause low turnover by overwhelming other processes. Pairwise comparisons with |RCbray| < 0.95 correspond to drift (undominated processes).

### 2.6. Statistical Analysis

Alpha diversity was calculated using Mothur (version 1.30.2), and Bray–Curtis was calculated from the relative abundances of OTUs using Fast Tree and UniFrac [64]. Permutational multivariate analysis of variance (PERMANOVA) was performed using the R vegan package [65] to assess the effects of the different treatments on changes in bacterial community composition. *p* < 0.05 (adjusted for false discovery rate) was considered significant for all statistical tests unless otherwise stated. Box plots, bar plots, and heatmaps were generated using R version 4.3.0 with the vegan, linkET, and ggplot2 packages [66].

## 3. Results

### 3.1. Warming Effects on Physical and Chemical Indicators

Water temperature in the mesocosms for the duration of the experiment followed the desired experimental design (Figure S1). In the constant warming treatment (T), the water temperature was consistently maintained at a level +4 °C higher than that of the control treatment (C). Meanwhile, in the heatwave treatment (H), the temperature exhibited fluctuations relative to the T treatment. Continuous monitoring revealed that the annual mean water temperature within the ambient temperature mesocosms was 19.5 °C, while both heated mesocosms maintained an identical average annual temperature of 23.5 °C, signifying a similar accumulation of water temperature.

**Figure 1:**
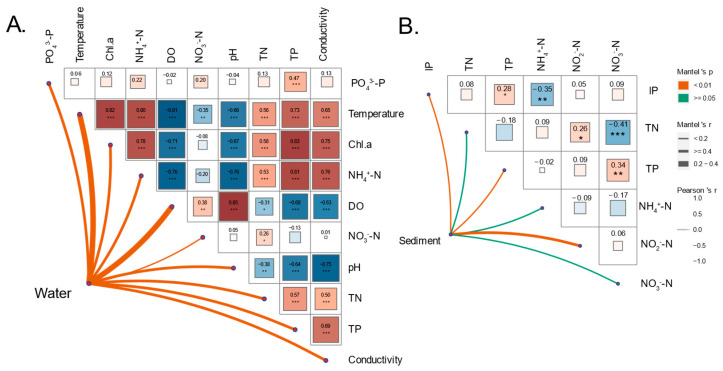
Heatmap shows correlations among environmental factors of water (**A**) and sediment (**B**) (Chl-a = chlorophyll-a, NH_4_^+^-N = ammonium, DO = dissolved oxygen, NO_3_^−^-N = nitrate, pH = pH value, TN = total nitrogen, TP = total phosphorus, IP = inorganic phosphate, NO_2_^−^-N = nitrite). Red squares indicate positive correlations, blue squares indicate negative correlations, and square size and color intensity reflect correlation strength. Values display Spearman correlation coefficients. * *p* < 0.05, ** *p* < 0.01, *** *p* < 0.001. Connecting lines depict associations between microbial communities and environmental factors in water and sediments (Mantel test, based on Bray–Curtis distance matrix). Red lines represent positive correlations, green lines represent negative correlations, and line thickness reflects correlation strength.

A set of environmental variables was monitored weekly (biweekly during the winter) throughout the experimental period (March 2018–February 2019) (Tables S1 and S2). It was observed that both the warming scenario and the changing seasons significantly impacted the physical and chemical indicators of water and sediment. Additionally, it was found that the season variation had a more pronounced effect on physico-chemical factors (Tables S3 and S4). The physico-chemical factor trends between seasons remained consistent across different treatments. Warming has a significant influence on several parameters in the water column, specifically conductivity, Chl-a, TN, and NH_4_^+^-N levels. During spring, summer, and autumn, warming led to elevated conductivity and TN levels, while winter experienced adverse effects. Furthermore, warming was associated with higher concentrations of both Chl-a and NH_4_^+^-N in the water column, with the impact being more significant in heatwaves (Table S1). Warming also has a notable impact on nitrogen levels in sediment, including NH_4_^+^-N, NO_3_^−^N, and TN. Interestingly, the TN content remained unaffected by seasonal variation. Warming led to an increase in the concentrations of both TN and NH_4_^+^-N in the sediment throughout the year. However, constant warming resulted in a significant decrease in NO_3_^−^-N concentration during summer and autumn, while heatwaves led to a decrease in NO_3_^−^-N concentration during summer but a significant increase in autumn and winter. Furthermore, water temperature was significantly correlated with all physico-chemical factors except for PO_4_^3-^-P. In sediments, TN showed a significant positive correlation with nitrite and a significant negative correlation with NO_3_^−^-N (Figure 1).

### 3.2. Bacterial Community Composition and Diversity in Water and Sediments

In this dynamic environment, we comprehensively analyzed microbial community composition using sequencing amplicon libraries. A total of 3,090,859 high-quality sequences were obtained from 72 water samples, while 2,603,551 high-quality sequences were obtained from 71 sediment samples. To account for the difference in sequencing depth, the planktonic and sedimentary bacterial sequences were normalized to 32,139 and 23,770, respectively, based on the minimum sequence number. Through clustering at a 97% sequence similarity level, we identified 4735 OTUs for the bacterioplankton and 12,825 OTUs for the sedimentary bacterial community. The rarefaction curves of water and sediment samples reached a plateau, indicating that the sequencing depth was sufficient and accurate (Figure S2).

**Figure 2:**
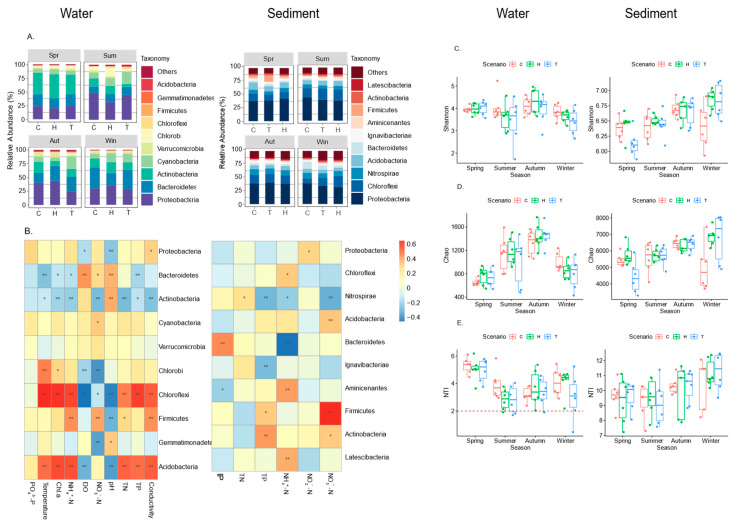
(**A**) The relative abundance of the top-10 phylum taxa of the bacterioplankton and sedimentary bacteria community. The presented relative abundances are based on the 97% similarity clusters of the OTUs. (**B**) Heatmaps of associations between top-10 phylum taxa and environmental factors. * *p* < 0.05, ** *p* < 0.01. (**C**–**E**) Shannon index, Chao index, and nearest-taxon index of bacterioplankton and sedimentary bacteria between treatments in different seasons.

In the water samples, a total of seven dominant phyla were identified, each accounting for a relative abundance of more than 1% at least in one sample. These seven phyla represented 97.07% of the sequences. In the sediment samples, a total of ten dominant phyla were identified, each accounting for a relative abundance of more than 1% in at least one sample, accounting for 87.11% of sequences. The phyla Proteobacteria (33.2% in water vs. 38.91% in sediments), Actinobacteria (21.01% in water vs. 1.84% in sediments), Chloroflexi (1.15% in water vs. 30.67% in sediments), Firmicutes (0.44% in water vs. 23.01% in sediments), and Acidobacteria (0.30% in water vs. 8.48% in sediments) were significantly abundant in both water and sediments (Figure 2A). The impact of warming observed at higher taxonomic levels was stronger on sedimentary bacteria than on planktonic bacteria (Tables S5 and S6). Specifically, in the sediment, compared with the control (ambient mesocosms), the relative abundances of Chloroflexi (Kruskal–Wallis test: *p* = 0.037), Aminicenantes (*p* = 0.030), and Latescibacteria (*p* = 0.023) significantly changed in the heatwave mesocosms, while the constant warming (T) mesocosms did not differ from the ambient mesocosms (Tables S7 and S8).

Dissimilarity tests confirmed significant differences between water and sediment communities, highlighting the distinct composition and structure of these microbial communities (Table S9). We observed significant influences of warming scenarios and seasonal variations on the bacterioplankton community. In contrast, no discernible differences were identified among the treatments concerning sedimentary bacteria (Table S10). To further investigate the underlying mechanisms that shape the microbial community in different habitats, a Mantel test was performed to correlate distance-corrected dissimilarities of the microbial community with those of environmental factors (Figure 1). The results revealed significant associations between the bacterioplankton community and all water properties, whereas the sedimentary bacterial community exhibited a significant association with NO_2_^−^-N, TP, and IP contents in sediment.

Bacterial community complexity was estimated using alpha diversity, including richness (Chao1 index) and diversity (Shannon index) (Table S11). The richness and diversity of sedimentary bacteria were significantly higher than those of bacterioplankton. Over time, there were no significant differences in the richness and diversity of bacterioplankton among the three temperature scenarios from spring to winter (*p* > 0.05, Wilcoxon test). However, we did observe notable differences in richness during spring between the ambient temperature group (C) and the heatwave group (H) of sedimentary bacteria. During winter, significant differences emerged in both diversity and richness between these two groups (*p* < 0.05) (Table S12).

Considering the treatment variations, the bacterioplankton diversity exhibits seasonal fluctuations, primarily with significant differences between the spring and autumn periods, as well as during winter. Additionally, bacterioplankton richness significantly increased (*p* < 0.001) from spring to autumn, followed by a decrease in winter (*p* < 0.001). In contrast, the richness and diversity of sedimentary bacteria exhibited distinct patterns, with changes becoming apparent from autumn onwards across all three temperature scenarios. Notably, the constant warming group exhibited a more pronounced alteration in richness and diversity compared to the other groups. During spring, the constant warming group showed relatively lower richness and diversity compared to the control and heatwave groups. However, as the season transitioned to winter, both the constant warming and heatwave groups displayed higher richness and diversity than the control group (Table S13; Figure 1A,B).

The NTI values for both water and sediment communities exhibited significant deviations from zero. Notably, sediment NTI values ranged from 7.22 to 12.47, significantly higher than water NTI values, which ranged from 0.46 to 6.19 (Figure 2C). Regarding the water body, a significant correlation between NTI and all physicochemical factors except PO_4_^3−^-P was observed (Table S14). However, no significant correlations were found between environmental factors and NTI values in sediments.

### 3.3. Co-Occurrence Patterns of Microbial Communities

**Figure 3:**
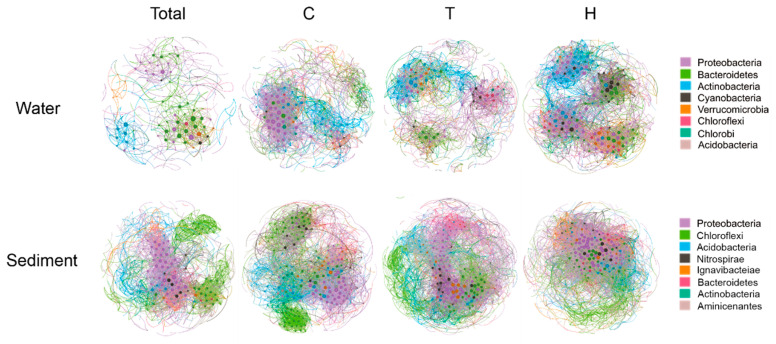
Co-occurrence networks of bacterioplankton and sedimentary bacteria of three treatments. (The nodes represent OTUs with a total abundance greater than 0.1% in samples. Spearman correlation ≥ │0.6│; *p* < 0.05). Node colors correspond to the phyla of the bacteria community. C, control; T, constant warming; H, heatwave.

Figure 3 presents co-occurrence networks for planktonic and sedimentary microbial communities using Spearman’s correlation coefficients. In these networks, nodes represent various bacterial phyla. In the planktonic bacteria community networks, the dominant phyla represented by the nodes include Proteobacteria, Bacteroidetes, Actinobacteria, Cyanobacteria, Verrucomicrobia, Chloroflexi, Chlorobi, and Acidobacteria. In the sedimentary bacteria community networks, the nodes represent the dominant phyla of Chloroflexi, Proteobacteria, Acidobacteria, Bacteroidetes, Verrucomicrobia, and Planctomycetes. Most edges in the network display positive correlations, indicating a predominantly cooperative relationship among microbial communities in both water and sediment. To assess the degree of species co-existence in different habitats, network topology parameters were calculated for each community group. The specific network topology parameters and their corresponding values are provided in Table 1 and Table S15. The network analysis revealed distinct co-occurrence patterns among different communities.

Specifically, our analysis revealed that the sediment network displayed a higher average degree and graph density, as well as a lower average path length, network diameter, modularity, and positive relationship compared to the water networks. These findings indicate that the benthic community of sedimentary bacteria is more interconnected, suggesting a higher frequency of co-occurrence among OTUs in sediment compared to planktonic bacteria communities. The higher co-occurrence frequency in sediment further underscores the interconnected nature of sedimentary microbial communities. Moreover, this study observed that the positive relationships within bacterioplankton networks were stronger than those in sedimentary bacteria networks, suggesting a more intense competition among species in sedimentary bacterial communities.

Furthermore, from a holistic perspective, the two warming scenarios both led to a significant increase in the network complexity of sedimentary bacteria when compared to the ambient control. This increase encompassed various aspects, such as network size, connectivity, average clustering coefficient, relative modularity, and the number of keystone species. On the contrary, there are differences in the impact of warming scenarios on the network topology structure of planktonic bacteria. When compared to the ambient warming group, we observed that the heatwave group exhibited a more interconnected network with a higher degree of clustering and transitivity. In contrast, the constant warming group had a less interconnected network with a lower degree of clustering and transitivity (Table 1). However, the effects of heatwaves and constant warming on bacterial networks vary across seasons (Tables S15 and S16). On a temporal scale, sedimentary and bacterioplankton networks exhibit seasonal changes. Bacterioplankton, especially, exhibit more diversity and complexity during summer and less during winter (Figure S3).

### 3.4. Disentangling Community Assembly Processes in Water and Sediments

**Figure 4:**
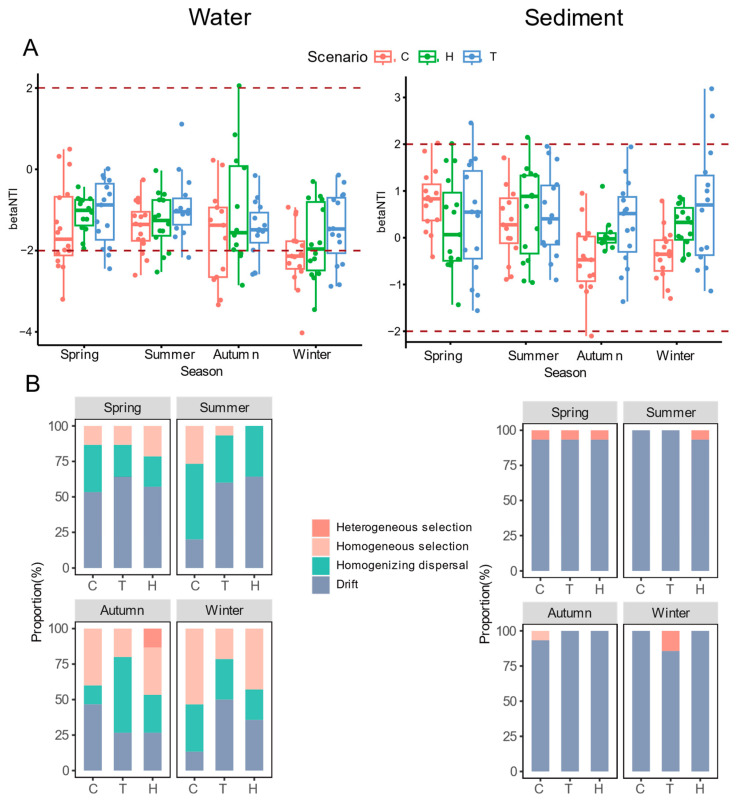
(**A**) Distribution of beta nearest taxon index (β-NTI) among bacterioplankton and sedimentary bacteria. (**B**) The proportion of heterogeneous selection, homogeneous selection, homogenizing dispersal, drift in bacterioplankton, and sedimentary bacteria assembly process.

The null model-based analyses were employed to explore the relative contributions of deterministic and stochastic processes to community assembly in water and sediments. βNTI values were calculated to assess the relative importance of two types of bacterial community assembly (deterministic and stochastic processes) in different habitats (Figure 4A). Most samples had βNTI values between −2 and 2, indicating that the stochastic process was the primary force influencing bacterial community assembly in all tested samples. Weighted βNTI and Bray–Curtis-based Raup–Crick (RCbray) were combined to approximate the relative contributions of different assembly processes, and null model analysis was conducted to estimate the ecological processes influencing the composition of bacterial communities.

For the benthic bacterial community, stochasticity emerged as the dominant ecological process, accounting for 85.71% to 100% of community turnover. This suggests that stochastic processes had a more significant influence on the assembly of this community. In fact, the undominated process (drift) accounted for 100%, indicating that no other ecological process played a more important role in driving the assembly of bacterial communities in sediment samples. On the other hand, the water community showed a relatively lower proportion of stochasticity (ranging from 46.67% to 100%), indicating that determinism played a relatively more substantial role in driving community assembly. These results revealed that the undominated stochastic process dominated the sediment communities. In contrast, homogenizing dispersal and homogeneous selection exerted a relatively greater influence on the water communities, leading to an increased similarity in planktonic bacterial communities over the course of the year.

The impact of warming on bacterial communities in the water column was apparent in the one-year study. However, sedimentary bacterial communities exhibited greater stability. In terms of seasonal variations throughout the year, both the ambient temperature group and the constant warming group exhibited a general trend of increasing importance of deterministic processes from spring to winter. Additionally, constant warming led to an increase in the proportion of stochastic processes throughout the entire year. However, the effects of heatwave on community assembly appeared to be more complex; it shows a decrease in deterministic processes in summer and an increase in autumn and winter (Figure 4B).

Overall, the analysis revealed distinct mechanisms governing the assembly of microbial communities in water and sediment communities, highlighting the context-dependent nature of these processes. To further understand the drivers of community dissimilarities, we examined the correlations between βNTI and environmental variables using Mantel tests (Table 2). The results revealed significant positive correlations between water community structure and environmental variables. Specifically, pH (r = 0.2039, *p* = 0.001), DO (r = 0.1067, *p* = 0.008), NH_4_^+^-N (r = 0.1620, *p* = 0.002), TN (r = 0.1261, *p* = 0.019), TP (r = 0.1160, *p* = 0.035), and Chl-a (r = 0.2213, *p* = 0.003) exhibited significant positive associations with the community structure. Moreover, sediment parameters such as TN (r = 0.1322, *p* = 0.014), NO_2_^−^-N (r = 0.1195, *p* = 0.029), and IP (r = 0.1180, *p* = 0.015) levels demonstrated significant positive correlations with βNTI.

## 4. Discussion

In this study, we conducted a comparative analysis of microbial interactions and community assembly mechanisms within mesocosms, focusing on water and sediment environments under various scenarios. Our results demonstrated the following: (1) bacterial communities in sediment exhibited higher levels of diversity, richness, stability, and phylogenetic clustering in comparison to the water body; (2) the dominant influence shaping both water and sediment communities was stochasticity, although determinism had a stronger influence in the water environment; and (3) constant warming increases the randomness of assembly among planktonic bacteria, whereas heatwaves present significant seasonal variations in this process.

### 4.1. Habitat Differences

Habitat differentiation likely contributed to differences in microbial community structure and assembly between lake water and sediments, and exploring the underlying ecological processes may be helpful in explaining these differences [28,67,68]. Consistent with previous research [39], our study showed that the sedimentary microbial communities exhibited higher taxonomic richness, diversity, and stronger phylogenetic clustering compared to their planktonic counterparts. The significant correlations observed between NTI values of planktonic bacterial communities and environmental factors suggested that the combination of these abiotic factors imposed a stronger pressure on bacterioplankton communities (Table S14).

Microbial correlation networks offer critical insights into the emergent properties of microbial communities. Our results, as depicted in Figure 3 and Table 1, revealed differences in network properties between water and sediment habitats. The more complex network in sediment suggested that the sedimentary network was more interconnected than the water network. Microbial interactions play a significant role in microbial community assembly by acting as a selection force, promoting niche differentiation, and fostering diversification [39]. Both the planktonic and sedimentary bacterial networks exhibited predominantly positive correlations, suggesting that the bacterial communities in water and sediments are primarily shaped by niche-sharing dynamics [53] and shared abiotic affinities. Organisms that share abiotic affinities have similar tolerances and requirements for factors such as temperature, humidity, pH, or nutrient availability. This leads to the establishment of mature microbial communities with niche separation and limited competition between microbes [69]. The percentage of positive correlations was higher in the planktonic bacterial network (97%) compared to the sedimentary bacterial network (83%), accompanied by higher diversity in sediments. A higher proportion of negative interactions within communities can enhance network stability under perturbations, indicating that the sedimentary bacterial network exhibits stronger interspecies competition compared to the planktonic bacterial network, potentially enhancing increased stability [70].

### 4.2. Assembly Process Differences

Phylogenetic null modeling analyses have suggested that stochastic assembly processes dominate in high-diversity communities [71]. Consistent with this perspective, sediment habitats are often characterized by higher heterogeneity and a wide range of microhabitats [39], as confirmed by our results showing greater diversity in sediment bacteria than planktonic bacteria. These microhabitats within sediments can create conditions distinct from the surrounding environment, providing refuge for species to survive in otherwise unsuitable conditions. Moreover, species residing in sediments can actively modify minerals and contribute to the formation of secondary minerals, further enhancing heterogeneity within sediments [72].

We find that the proportion of stochastic processes in sediments was higher compared to the water body. In sedimentary bacteria, drift dominates, while in planktonic bacteria, the random processes are jointly dominated by drift and homogenizing dispersal. Drift refers to the random changes in the relative abundance of different species due to random births, deaths, and reproduction events. Homogenizing dispersal, on the other hand, refers to the movement of organisms between different habitats, leading to a more uniform distribution of species across habitats [73]. Planktonic bacteria within the aquatic environment exhibit a tendency towards dispersal. According to the spatial insurance hypothesis, high dispersal levels aid in the resistance of bacterioplankton against the impacts of heatwaves and warming [74]. Dispersal allows species to move between different habitats, which can help maintain diversity and ecosystem functioning in changing environments [75]. This difference in the dominant processes shaping the communities may be attributed to the distinct environmental conditions and spatial structures of sediments and water bodies. Sediments are more heterogeneous environments, with higher stability and less affected by abiotic factors, compared to water bodies, which are more evenly mixed during hydrodynamic movements [76]. When environmental conditions support the coexistence of multiple species, the community experiences reduced selection pressure, and stochastic processes primarily govern the assembly [77,78]. Drift can play a significant role in maintaining diversity in systems with high selection pressures and low dispersal [79]. However, due to their small size, high abundance, and short generation time, microorganisms have not been extensively studied or quantified in terms of dispersal processes. Microbial communities in water exhibit spatial and temporal differences [80,81], which in turn influence dispersal patterns.

In this study, it was found that the proportion of different assembly processes in the assembly of planktonic bacteria varies greatly. Mantel tests showed that a suite of environmental variables had a positive association with water community assembly (Table 2). Temperature directly influences assembly; instead, constant warming and heatwaves have varying indirect effects on the assembly of planktonic bacterial communities (Figure 4B). Abiotic factors, such as temperature, can indirectly influence the ecological processes underlying community assembly in water. Environmental factors can drive changes in microbial communities, affecting their diversity and composition [82]. In some cases, temperature can indirectly affect microbial community assembly by altering other environmental factors, such as nutrient availability or water chemistry [83].

### 4.3. The Impact of Seasonal Change and Warming

We find that seasonal variations had a more pronounced influence on microbial diversity and community composition in both water and sediment compared to temperature treatments, indicating the robustness of yearly rhythms in microbial communities. Seasonality plays a crucial role in shaping the temporal patterns of bacterial community composition in natural environments [84,85]. In natural aquatic ecosystems, microbial communities often exhibit seasonal shifts in their composition, activity, and function [68]. Studies have shown that sporadic meteorological events and irregular nutrient supply in coastal areas do not significantly affect seasonal patterns [86].

Homogenizing dispersal typically manifests in communities inhabiting relatively stable and small environments [87]. In our study, we did not observe significant variations in bacterioplankton diversity and richness among different warming scenarios across all seasons. The lack of pronounced differences could potentially be ascribed to the high dispersal rate of bacteria, which may have obscured our ability to detect changes in alpha diversity. Under prolonged exposure to high temperatures, we observed changes in community composition and assembly; bacterial communities may shift towards heat- or heatwave-resistant communities [88,89,90]. In autumn, the planktonic bacteria within the constant warming group displayed a comparatively stable community structure when contrasted with the ambient temperature group, as they exhibited a higher degree of homogenizing dispersal.

In this experiment, we observed that both warming scenarios enhance sedimentary microbial network complexity and stability. Molecular ecological networks under warming became significantly more robust, with network stability strongly correlated with network complexity, supporting the central ecological belief that complexity begets stability [91]. However, the effects of warming on microbial communities in aquatic environments are not uniform and may be influenced by various factors, including trophic conditions. Similar to sedimentary bacterial communities, heatwaves have been observed to augment the complexity and stability of planktonic bacterial networks. However, the impact of heatwave on the assembly process exhibits season-dependent variability. Change, often perceived as indicative of vulnerability and instability in a system, can alternatively signify a higher adaptability level [92]. This dual perspective applies to bacterial planktonic communities, demonstrating significant adaptability to environmental changes despite potential stress and disturbance [93]. While the functional response to heatwave disturbances may initially be compromised, the multivariate composition of the community exhibits a slower recovery, highlighting challenges to stability [94]. The occurrence of heatwaves poses unforeseen challenges, necessitating further adaptation for efficient stressor coping. Consequently, adapting to heatwaves proves to be a complex task for bacterial planktonic communities. Constant warming throughout the year seems to introduce stochasticity into the assembly dynamics of planktonic bacterial communities. This stochasticity is characterized by a reduction in interconnections within the planktonic bacterial network, diminished clustering, and a sparser network structure [95]. Consequently, these alterations result in heightened intensity and frequency of interactions among bacterial species. These findings underscore the concept that environmental shifts, such as escalating temperatures, have the potential to induce substantial structural and functional modifications within planktonic bacterial communities. This heightened stochasticity within the planktonic bacterial community may result in increased instability in both community structure and function. Such increased instability has the potential to negatively impact the overall stability and resilience of the ecosystem, potentially reducing its capacity to adapt to environmental disturbances. Furthermore, the intensified interactions among bacterial species can alter the dominant position of certain taxa within the community, thereby influencing the ecosystem’s overall functioning and stability [95,96].

Heatwaves were found to stimulate an increase in bacterial abundance, and this effect was more pronounced in sediment [97]. The richer substrate and higher relative temperature increase (from 7 °C to 11 °C) in the sediment would facilitate a stronger warming effect on microbial abundance during colder winters. Given the likely differences in nutrient concentrations between sediment and water, it is plausible to hypothesize that the divergent responses to warming in these habitats may be attributed to contrasting nutrient availability. Previous studies have indicated that nutrient enrichment can interact with warming to drive shifts in bacterioplankton communities [40]. Notably, warming has more pronounced effects in colder regions compared to warmer ones [98,99]. During colder seasons, heatwaves have a substantial impact on the composition, richness, and diversity of sediment bacterial communities, while their influence on assembly processes is comparatively minor. We find heatwaves significantly impact the relative abundance of Firmicutes of water in cold seasons. The heatwave significantly impacted Chloroflexi, Aminicenantes, Bacteroidetes, and Actinobacteria in the sediment. Warming has long been observed to increase the abundance of Gram-positive bacteria, particularly Actinobacteria [100]. Analysis of contigs and MAGs indicated that Actinobacteria might hold a competitive edge during extreme summers because of the production of geosmin and 2-methylisoborneol [101]. Higher temperatures may enhance the speed of nitrification and denitrification [102]. In our study, warming has a significant impact on the levels of TN, NO_3_^−^-N, and NH_4_^+^-N. Aminicenantes are involved in processes such as ammonia oxidation and denitrification, leading to notable fluctuations in their abundance during cold seasons.

In contrast, we did not observe differences in sediment bacterial community assembly patterns between seasons and warming scenarios. This can be attributed to the nature of the sediment habitat. The more clustered network structure and stronger species connections in sediments could enhance resource and information transfer efficiency, resulting in high stability of community function. Sedimentary bacterial communities are known to be more persistent and have limited effects on planktonic bacteria in the water column of long-term stable ecosystems, primarily due to the habitat-specific bacterial community compositions [40,103,104].

## 5. Conclusions

In this year-long study, we investigated the effects of constant warming and heatwaves on bacterial communities in water bodies and sediment across all four seasons. Our findings reveal that warming scenarios significantly contribute to seasonal variations in community composition, but long-term warming is insufficient to overwhelm the influence of seasonal variation. Notably, we observed an increase in deterministic processes during specific seasons in the heatwave group compared to the ambient temperature group, signifying a shift toward more predictable assembly dynamics. This underscores the dynamic interplay between warming scenarios and seasonal influences on bacterial communities. However, constant warming led to an increase in the random process of planktonic bacterial communities across all seasons. Furthermore, we determined that planktonic bacterial assembly is primarily driven by homogenization, while sedimentary bacterial assembly is predominantly influenced by drift. This research advances our understanding of how climate change affects bacterial communities, providing insights into the intricate dynamics within aquatic ecosystems.

## Figures and Tables

**Table 1:** Topological properties of co-occurrence networks for bacterioplankton and sedimentary bacteria.

Habitat	Scenario	Nodes	Edges	Average Degree	Average Transitivity	Average Clustering Coefficient	Average Path Length	Diameter	Modularity	Graph Density	Positive Relationship (%)
Water	Total	316	892	5.646	0.390	0.458	3.614	10.816	0.053	0.018	97.646
	C	325	2012	12.382	0.487	0.502	1.230	6.838	0.009	0.038	82.604
	T	351	1720	9.801	0.461	0.491	2.096	8.295	0.021	0.028	93.953
	H	360	2962	16.456	0.520	0.550	0.994	5.170	0.003	0.046	86.124
Sediment	Total	831	9531	22.939	0.507	0.610	1.501	7.148	0.005	0.028	87.483
	C	655	8451	25.805	0.456	0.472	0.471	4.438	0.0005	0.039	71.459
	T	704	10,006	28.426	0.508	0.479	0.459	6.127	0.001	0.040	77.294
	H	621	12,642	40.715	0.526	0.520	0.456	5.804	0.001	0.066	57.475

**Table 2:** Mantel tests for the correlation between environmental variables and βNTI of bacterial communities in water and sediment.

	Water			Sediment	
	r	*p*		r	*p*
Temperature	0.0369	0.219	TN	0.1322	0.014 *
pH	0.2039	0.001 ***	NH_4_^+^-N	0.0020	0.432
DO	0.1067	0.008 *	NO_2_^−^-N	0.1195	0.029 *
Conductivity	0.0259	0.001 ***	NO_3_^−^-N	−0.0110	0.536
NO_3_^−^-N	−0.0429	0.759	TP	0.0853	0.063
NH_4_^+^-N	0.1620	0.002 **	IP	0.1180	0.015 *
TN	0.0146	0.019 *			
PO_4_^3−^-P	0.0663	0.194			
TP	0.1160	0.035 *			
Chl-a	0.2213	0.003 **			

* *p* < 0.05, ** *p* < 0.01, *** *p* < 0.001.

## Data Availability

Our paired-end Illumina sequence data used in summer are available in the Sequence Read Archive (SRA) of the National Center for Biotechnology Information (NCBI), BioProject accession number PRJNA705584. The paired-end Illumina sequence data in spring and autumn have not been published.

## Supplementary Materials

The following supporting information can be downloaded at https://www.mdpi.com/article/10.3390/microorganisms11112807/s1, Table S1: Physicochemical properties of water during experiment; Table S2: Physicochemical properties of sediments for this study; Table S3: Results of PERMANOVA tests (Euclidean dissimilarity) testing the effects of warming and treatment on physicochemical properties in water throughout the experiment; Table S4: Results of PERMANOVA tests (Euclidean dissimilarity) testing the effects of warming and treatment on physicochemical properties in sediments over the study duration; Table S5: Results of PERMANOVA tests (Euclidean dissimilarity) testing the effects of warming and treatment on the top-10 phylum taxa of the bacteria communities in water; Table S6: Results of PERMANOVA tests (Euclidean dissimilarity) testing the effects of warming and treatment on the top-10 phylum taxa of the bacteria communities in sediments; Table S7: Results of Kruskal–Wallis test testing the effects of different temperature treatment on the top-10 phylum taxa of the bacteria communities in water; Table S8: Results of Kruskal–Wallis test testing the effects of different temperature treatment on the top-10 phylum taxa of the bacteria communities in sediment; Table S9: Dissimilarity tests of microbial communities for water and sediments. The values of upper rows for MRPP, ANOSIM, and PERMANOVA are delta, R-value, and F-value, respectively. The values of lower rows are the significance value (*p*-value); Table S10: Permutational multivariate analysis of variance (PERMANOVA) based on incidence-based Bray–Curtis dissimilarity in bacterioplankton and sedimentary bacteria community composition in experimental mesocosms characterized by different warming and seasons; Table S11: Alpha diversity of bacteria in water and sediment; Table S12: Results of Wilcoxon tests on the effects of different scenarios on alpha diversity; Table S13: Results of Wilcoxon tests on the effects of different seasons on alpha diversity; Table S14: Relationships between within-community NTI and environmental factors in the water and sediment (based on general linear model); Table S15: Topological properties of co-occurrence network for bacterioplankton and sedimentary bacteria; Table S16: Topological properties of the planktonic and sedimentary bacterial networks. Figure S1: Temperature trend for all the treatments during the experiment. Each point on the curve represents the average temperature of the day. C, environment temperature; T, constant warming; H, heatwave. Figure S2: Rarefaction curves of water and sediments constructed based on the number of reads sampled and the number of OTUS. Figure S3: Co-occurrence network of bacterioplankton and sedimentary bacteria of four seasons. (The nodes represent OTUs with a total abundance greater than 0.1% in samples. Spearman correlation ≥ |0.6|; *p* < 0.05.) Node colors correspond to the phyla of the bacteria community.
