# Supplementary material for: The Impact of Warming on Assembly Processes and Diversity Patterns of Bacterial Communities in Mesocosms
Source: Microorganisms. 2023 Nov 19;11(11):2807. doi: 10.3390/microorganisms11112807 (PMC10672829; doi:10.3390/microorganisms11112807)
Supplement: Supplementary file 1 [file microorganisms-11-02807-s001.zip › microorganisms-2686399-supplementary.pdf]

Table S1. Physicochemical properties of water during experiment.

Season	Treatment	pH	DO (mg/L)	Temperature (°C)	Conductivity (μS/cm)	Chl-a (μg/L)	TN (mg/L)	NH_4^+-N (mg/L)	NO_3^--N (mg/L)	TP (mg/L)	PO_4^{3-}-P (μg/L)
Spring	C	9.89±0.27	8.56±1.18	20.14	191.13±5.43	2.43±1.53	0.51±0.23	0.05±0.00	0.04±0.02	0.04±0.01	13.22±4.29
	T	9.29±0.38	7.75±0.47	24.19	225.09±8.29	5.81±4.70	0.59±0.19	0.14±0.06	0.02±0.01	0.06±0.03	16.16±0.55
	H	9.15±0.24	7.39±0.66	24.27	222.57±10.40	3.69±1.35	0.55±0.01	0.09±0.01	0.01±0.00	0.03±0.01	14.51±4.87
Summer	C	7.67±0.67	1.99±2.37	29.59	217.50±26.78	9.30±5.62	0.85±0.27	0.15±0.02	0.07±0.02	0.09±0.02	18.47±9.06
	T	8.35±0.97	3.53±3.61	33.71	275.40±42.04	31.55±10.03	1.24±0.51	0.17±0.05	0.15±0.04	0.12±0.05	31.95±25.94
	H	7.70±0.27	1.91±1.96	33.99	306.64±5.09	42.82±10.30	1.27±0.33	0.25±0.07	0.12±0.01	0.16±0.10	24.81±24.06
Autumn	C	7.63±0.26	4.52±1.73	20.86	264.81±31.63	11.03±4.93	0.58±0.14	0.14±0.03	0.14±0.07	0.12±0.03	24.27±12.46
	T	8.17±0.47	7.64±0.71	24.81	307.08±46.55	33.61±16.43	1.33±0.40	0.23±0.07	0.18±0.01	0.14±0.03	16.73±7.59
	H	7.88±0.28	4.45±2.43	24.63	324.27±39.70	23.44±9.05	1.02±0.12	0.21±0.07	0.13±0.05	0.15±0.07	22.76±10.70
Winter	C	9.40±0.13	13.49±0.30	7.18	209.90±3.40	1.29±0.23	0.70±0.08	0.03±0.01	0.39±0.08	0.01±0.002	--
	T	9.71±0.19	12.67±0.89	11.13	203.80±3.46	2.89±1.68	0.60±0.06	0.02±0.01	0.22±0.05	0.02±0.01	--
	H	9.48±0.16	13.02±0.83	10.77	207.30±3.58	1.72±0.18	0.67±0.16	0.06±0.04	0.28±0.05	0.02±0.002	--

Note: --, not detected.

Table S2. Physicochemical properties of sediments for this study.

Season	Treatment	IP (mg/kg)	TP (g/kg)	TN (g/kg)	NH_4^+-N (mg/kg)	NO_3^--N (mg/kg)	NO_2^--N (mg/kg)
Spring	C	0.91±0.21	1.05±0.25	3.19±0.26	72.12±6.39	2479.47±328.90	102.41±20.36
	T	0.92±0.30	1.22±0.35	3.45±0.36	66.99±22.52	2663.68±456.05	98.50±25.16
	H	0.85±0.07	0.76±0.27	3.72±0.06	71.46±6.62	2837.82±334.87	110.88±20.29
Summer	C	0.84±0.20	1.06±0.16	3.21±0.18	73.55±76.98	1761.95±98.53	134.89±13.66
	T	1.01±0.14	0.95±0.17	3.83±0.07	125.98±52.98	259.81±152.01	119.64±7.74
	H	0.78±0.18	0.96±0.13	3.47±0.19	141.59±55.67	347.77±232.74	86.69±37.45
Autumn	C	0.83±0.59	0.58±0.29	3.58±0.74	50.46±58.08	105.10±44.30	125.23±42.43
	T	1.01±0.62	0.76±0.36	3.93±0.14	110.09±50.54	52.39±42.11	102.26±16.45
	H	0.69±0.37	0.75±0.24	3.69±0.22	169.24±27.89	425.62±109.80	111.62±43.19
Winter	C	1.18±0.19	0.90±0.37	3.70±0.52	26.94±12.56	529.46±295.32	102.67±12.81
	T	1.49±0.04	1.01±0.23	3.66±0.25	70.22±58.77	869.75±278.94	138.28±53.60
	H	1.22±0.10	1.08±0.22	3.57±0.24	53.18±23.75	1239.36±196.92	97.89±6.78

Table S3. Results of PERMANOVA tests (Euclidean dissimilarity) testing the effects of warming and treatment on physicochemical properties in water throughout the experiment.

	Temperature	pH	Conductivity	Chl-a	DO	TN	NH_4^+-N	NO_3^--N	TP	PO_4^{3-}-P
Warming	0.03*	0.129	0.001***	0.001***	0.252	0.007**	0.008**	0.721	0.075	0.826
Season	0.001***	0.001***	0.001***	0.001***	0.001***	0.001***	0.001**	0.002**	0.001***	0.313
Warming: Season	0.994	0.45	0.003**	0.016*	0.194	0.002**	0.116	0.056	0.514	0.897

* p <0.05, ** p <0.01, *** p <0.001

Table S4. Results of PERMANOVA tests (Euclidean dissimilarity) testing the effects of warming and treatment on physicochemical properties in sediments over the study duration.

	IP	NH_4^+-N	NO_2^--N	NO_3^--N	TN	TP
Warming	0.052	0.002**	0.147	0.001***	0.004**	0.75
Season	0.001***	0.002**	0.842	0.001***	0.092	0.002**
Warming: Season	0.965	0.087	0.061	0.001***	0.108	0.153

* p <0.05, ** p <0.01, *** p <0.001

Table S5. Results of PERMANOVA tests (Euclidean dissimilarity) testing the effects of warming and treatment on the top-10 phylum taxa of the bacteria communities in water.

	Treatment	Season	Treatment: Season
Proteobacteria	0.499	0.005**	0.177
Bacteroidetes	0.559	0.003**	0.397
Actinobacteria	0.121	0.001***	0.227
Cyanobacteria	0.115	0.243	0.574
Verrucomicrobia	0.723	0.103	0.590
Chlorobi	0.540	0.001***	0.187
Chloroflexi	0.254	0.001***	0.472
Firmicutes	0.395	0.001***	0.822
Gemmatimonadetes	0.380	0.001***	0.021*
Acidobacteria	0.381	0.002**	0.299
Others	0.958	0.003**	0.999

* $p < 0.05$, ** $p < 0.01$, *** $p < 0.001$

Table S6. Results of PERMANOVA tests (Euclidean dissimilarity) testing the effects of warming and treatment on the top-10 phylum taxa of the bacteria communities in sediments.

	Treatment	Season	Treatment: Season
Proteobacteria	0.387	0.094	0.323
Chloroflexi	0.009**	0.001***	0.006**
Nitrospirae	0.013*	0.001***	0.309
Acidobacteria	0.557	0.001***	0.383
Bacteroidetes	0.002**	0.001***	0.009**
Ignavibacteriae	0.225	0.001***	0.08
Aminicenantes	0.063	0.007**	0.338
Firmicutes	0.001***	0.001***	0.001***
Actinobacteria	0.003**	0.001***	0.009**
Latescibacteria	0.068	0.517	0.809
Others	0.809	0.001***	0.329

* p <0.05, ** p <0.01, *** p <0.001

Table S7. Results of Kruskal-Wallis test testing the effects of different temperature treatments on the top-10 phylum taxa of the bacteria communities in water.

	Total			Spring			Summer			Autumn			Winter		
	C-T	C-H	T-H	C-T	C-H	T-H	C-T	C-H	T-H	C-T	C-H	T-H	C-T	C-H	T-H
Proteobacteria	0.212	0.869	0.266	0.873	0.262	0.262	0.631	0.262	0.522	0.037*	0.631	0.037*	0.936	0.109	0.150
Bacteroidetes	0.885	0.635	0.523	0.631	0.337	0.522	0.749	0.522	0.749	0.423	0.150	0.423	0.337	0.055	0.200
Actinobacteria	0.132	0.503	0.303	0.749	0.423	0.200	0.025*	0.873	0.150	0.200	0.004**	0.423	0.631	0.631	0.423
Cyanobacteria	0.201	0.665	0.398	0.423	0.873	0.522	0.337	0.423	0.873	0.055	0.200	0.006**	0.262	0.262	0.873
Verrucomicrobia	0.244	0.951	0.409	0.873	0.200	0.200	0.109	0.631	0.337	0.749	0.150	0.055	1.000	0.749	0.873
Chlorobi	0.152	0.726	0.327	0.016*	0.150	0.631	0.749	0.522	0.200	0.055	0.873	0.016*	0.749	0.004**	0.064
Chloroflexi	0.523	0.959	0.695	0.262	0.262	1.000	0.337	0.200	0.873	0.109	0.200	0.337	0.334	0.128	0.810
Firmicutes	0.749	0.951	0.703	0.258	0.024*	0.337	0.749	0.873	0.749	1.000	0.749	0.749	0.522	0.025*	0.173
Gemmatimonadetes	0.557	0.557	0.773	0.016*	0.055	0.873	0.077	0.688	0.197	0.936	0.936	0.520	0.872	0.172	0.378
Acidobacteria	0.19	0.312	0.741	0.199	0.423	0.936	0.025*	0.337	0.150	0.336	0.261	0.873	0.521	0.873	0.335
Others	0.741	0.918	0.703	0.631	0.337	0.749	0.749	0.749	0.873	0.522	0.631	0.873	0.522	0.749	0.423

* $p < 0.05$, ** $p < 0.01$, *** $p < 0.001$

Table S8. Results of Kruskal-Wallis test testing the effects of different temperature treatments on the top-10 phylum taxa of the bacteria communities in sediment.

	Total			Spring			Summer			Autumn			Winter		
	C-T	C-H	T-H	C-T	C-H	T-H	C-T	C-H	T-H	C-T	C-H	T-H	C-T	C-H	T-H
Proteobacteria	0.509	0.264	0.546	0.873	0.078	0.200	0.423	0.378	0.873	0.873	0.715	0.273	0.273	0.055	0.273
Chloroflexi	0.670	0.037*	0.070	0.262	0.631	0.337	0.522	0.522	0.749	0.873	1.000	0.855	0.201	0.006**	0.018*
Nitrospirae	0.077	0.120	0.560	0.200	0.423	0.016*	0.522	0.262	0.749	0.262	0.018*	0.100	0.201	0.200	0.855
Acidobacteria	0.360	0.898	0.448	0.109	0.200	0.522	1.000	0.423	0.262	1.000	1.000	0.584	0.584	0.262	0.855
Bacteroidetes	0.610	0.061	0.263	0.749	0.262	0.423	0.150	0.262	0.575	1.000	0.068	0.100	0.465	0.025*	0.045*
Ignavibacteriae	0.865	0.242	0.199	0.873	0.055	0.037*	0.873	0.423	0.337	0.873	0.855	0.584	0.855	0.055	0.144
Aminicenantes	0.750	0.030*	0.109	0.150	1.000	0.173	0.262	0.423	1.000	0.631	0.584	0.855	0.715	0.045*	0.100
Firmicutes	0.395	0.573	0.144	0.055	0.423	0.025*	1.000	0.688	0.749	0.200	0.855	0.201	0.855	0.078	0.018*
Actinobacteria	0.194	0.307	0.051	0.037*	0.016*	0.004**	0.936	0.423	0.873	0.688	0.201	1.000	0.201	0.748	0.169
Latescibacteria	0.873	0.023*	0.060	0.109	0.688	0.109	0.470	0.262	0.631	0.522	0.361	0.465	0.465	0.150	0.361
Others	0.407	0.033*	0.339	0.873	0.262	0.873	0.522	0.521	0.748	0.749	0.273	0.100	0.100	0.037*	1.000

* p <0.05, ** p <0.01, *** p <0.001

Table S9. Dissimilarity tests of microbial communities for water and sediments. The values of upper rows for MRPP, ANOSIM, and PERMANOVA are delta, R value, and F-value, respectively. The values of lower rows are the significance value (*P-value*).

Water-Sediment	MRPP	ANOSIM	PERMANOVA
Bray-Curtis	0.8126	0.9752	80.5479
	0.0010	0.0010	0.0010
Jaccard	0.8730	0.9752	47.4635
	0.0010	0.0010	0.0010

Table S10. Permutational multivariate analysis of variance (PERMANOVA) based on incidence-based Bray-Curtis's dissimilarity in bacterioplankton and sedimentary bacteria community composition in experimental mesocosms characterized by different warming and seasons.

Habitat		d.f.	R^2	F model	*p*
Water	Warming	2	0.036	1.710	0.003**
	Season	3	0.244	7.786	0.001***
	Warming: Season	6	0.094	1.500	0.002**
	Residuals	60	0.626		
Sediment	Warming	2	0.028	0.983	0.467
	Season	3	0.043	0.996	0.434
	Warming: Season	6	0.089	1.035	0.378
	Residuals	59	0.841		

* p <0.05, ** p <0.01, *** p <0.001

Significance tests were performed using F-tests based on sequential sums of squares from permutations of the raw data.

Table S11. Alpha diversity of bacteria in water and sediment.

Season	Scenario	Water		Sediment	
		Shannon	Chao1	Shannon	Chao1
Spring	C	5.71 ±0.18	620.24 ±62.54	9.17 ±0.23	5029.65 ±296.04
	T	5.80 ±0.45	645.93 ±161.82	8.82 ±0.31	3969.77 ±939.44
	H	5.72 ±0.37	686.06 ±175.62	9.26 ±0.28	5259.29 ±539.14
Summer	C	5.79 ±0.89	1124.86 ±320.06	9.20 ±0.31	5079.52 ±809.88
	T	4.93 ±1.44	940.69 ±383.35	9.25 ±0.30	5097.21 ±457.13
	H	5.18 ±0.88	1124.36 ±198.57	9.35 ±0.11	5221.79 ±180.54
Autumn	C	5.90 ±0.57	1261.08 ±233.55	9.59 ±0.20	5797.61 ±85.67
	T	5.72 ±0.88	1378.92 ±243.45	9.51 ±0.31	5510.96 ±403.77
	H	6.21 ±0.85	1370.31 ±210.53	9.58 ±0.30	5597.36 ±339.00
Winter	C	5.53 ±0.49	904.55 ±178.19	9.19 ±0.49	4085.34 ±1115.94
	T	4.92 ±0.83	719.45 ±200.08	9.78 ±0.40	5549.74 ±1223.17
	H	5.26 ±0.39	783.60 ±134.71	9.80 ±0.24	5778.45 ±415.63

Table S12. Results of Wilcoxon tests on the effects of different scenarios on alpha-diversity.

		Water		Sediment	
		Chao1 *p-value*	Shannon *p-value*	Chao1 *p-value*	Shannon *p-value*
total	C-H	1	0.791	0.106	0.438
total	C-T	1	1	1	1
total	H-T	1	1	0.828	0.695
spring	C-H	0.699	0.937	0.041*	0.093
spring	C-T	0.589	0.589	0.24	0.31
spring	H-T	0.394	1	0.065	0.065
summer	C-H	0.937	0.31	0.589	0.937
summer	C-T	0.699	0.937	0.818	0.394
summer	H-T	0.589	0.485	0.937	0.699
autumn	C-H	0.937	0.394	0.931	0.792
autumn	C-T	0.589	0.485	0.937	1
autumn	H-T	0.699	0.937	0.662	1
winter	C-H	0.589	0.394	0.015*	0.041*
winter	C-T	0.589	0.18	0.065	0.093
winter	H-T	0.818	0.589	1	1

Table S13. Results of Wilcoxon tests on the effects of different seasons on alpha-diversity.

			Water		Sediment	
			Chao1	Shannon	Chao1	Shannon
			p-value	*p-value*	*p-value*	*p-value*
total	spring	summer	0.001***	0.089	0.256	0.205
total	spring	autumn	0.001***	0.509	0.003**	0.0003***
total	spring	winter	0.001***	0.008*	0.221	0.001**
total	summer	autumn	0.003**	0.089	0.005**	0.005**
total	summer	winter	0.024*	0.743	0.335	0.007**
total	autumn	winter	0.001***	0.024*	0.782	0.613
C	spring	summer	0.39	0.589	0.240	1
C	spring	autumn	0.009**	0.818	0.041*	0.180
C	summer	autumn	0.002**	0.818	0.065	0.065
C	winter	spring	0.699	0.937	0.132	0.818
C	winter	summer	0.041*	0.310	0.699	0.937
C	winter	autumn	0.002**	0.310	0.009**	0.009**
T	spring	summer	0.240	0.310	0.065	0.065
T	spring	autumn	0.002**	0.937	0.009**	0.009**
T	summer	autumn	0.015*	0.394	0.180	0.310
T	winter	spring	0.485	0.041*	0.026*	0.004**
T	winter	summer	0.180	0.937	0.394	0.026*
T	winter	autumn	0.002**	0.132	0.937	0.394
H	spring	summer	0.002**	0.093	0.177	0.177
H	spring	autumn	0.002**	0.699	0.002**	0.004**
H	summer	autumn	0.065	0.065	0.082	0.247
H	winter	spring	0.093	0.065	0.093	0.009**
H	winter	summer	0.026*	0.589	0.537	0.126
H	winter	autumn	0.002**	0.180	0.589	0.699

Table S14. Relationships between within-community NTI and environmental factors in the water and sediment (based on general linear model).

	Water			Sediment	
	Correlation	*p-value*		Correlation	*p-value*
Temperature	-0.283	0.020*	TN	-0.055	0.668
pH	0.370	0.002**	NH_4^+-N	0.050	0.697
DO	0.241	0.049*	NO_2^--N	0.231	0.067
Conductivity	-0.395	0.001***	NO_3^--N	-0.030	0.814
Chl-a	-0.401	0.001***	TP	-0.010	0.938
TN	-0.320	0.008**	IP	-0.074	0.560
NO_3^--N	-0.353	0.004**			
NH_4^+-N	-0.318	0.012*			
PO_4^{3-}-P	-0.246	0.082			
TP	-0.374	0.002**			

Table S15. Topological properties of co-occurrence network for bacterioplankton and sedimentary bacteria.

Habitat	Season	Nodes	Edges	Average Degree	Average transitivity	Average Clustering coefficient	Average path length	Diameter	Modularity	Graph density	Positive Relationship (%)
Water	Spring	130	297	4.569	0.468	0.510	1.462	6.179	0.111	0.035	73.401
	Summer	307	1088	7.088	0.364	0.375	1.873	9.808	0.006	0.023	80.882
	Autumn	166	222	2.675	0.396	0.359	3.099	8.701	0.608	0.016	90.541
	Winter	69	41	1.188	0	0	1.112	3.332	0.947	0.017	87.805
Sediment	Spring	529	7338	27.743	0.486	0.461	0.259	2.933	0.000	0.053	64.282
	Summer	509	3624	14.240	0.505	0.410	0.597	5.939	0.009	0.028	66.253
	Autumn	457	3500	15.317	0.468	0.411	0.258	3.075	0.002	0.034	59.171
	Winter	557	12616	45.300	0.717	0.543	0.699	5.297	0.001	0.081	63.079

Table S16. Topological properties of the planktonic and sedimentary bacterial networks.

Habitat	Season	Scenario	Nodes	Edges	Average Degree	Average transitivity	Average Clustering coefficient	Average path length	Diameter	Modularity	Graph density	Positive Relationship (%)
Water	Spring	C	38	25	1.316	1	1	0.48	1	0.922	0.036	60
	Spring	T	24	15	1.25	1	1	0.733	1	0.88	0.054	80
	Spring	H	43	29	1.349	1	1	0.517	1	0.93	0.032	62.069
	Summer	C	98	136	2.776	0.958	0.884	0.759	2.957	0.911	0.029	85.294
	Summer	T	77	107	2.779	0.79	0.839	0.771	3.941	0.76	0.037	79.439
	Summer	H	28	14	1	NA	NA	0.857	1	0.929	0.037	85.714
	Autumn	C	45	24	1.067	1	1	0.917	1	0.948	0.024	91.667
	Autumn	T	33	24	1.455	1	1	1	1	0.802	0.045	100
	Autumn	H	107	119	2.224	0.807	0.874	1.039	2.957	0.868	0.021	78.992
	Winter	C	23	13	1.13	1	1	0.692	1	0.888	0.051	69.231
	Winter	T	71	76	2.141	0.9	0.894	0.892	2.956	0.904	0.031	86.842
	Winter	H	29	16	1.103	1	1	0.75	1	0.914	0.039	81.25
Sediment	Spring	C	133	181	2.722	0.909	0.894	0.413	2.957	0.922	0.021	61.878
	Spring	T	152	277	3.645	0.858	0.891	0.423	1.971	0.849	0.024	63.538
	Spring	H	137	163	2.38	0.856	0.796	0.674	4.928	0.895	0.017	69.325
	Summer	C	135	332	4.919	0.828	0.855	0.394	2.957	0.797	0.037	59.337
	Summer	T	127	280	4.409	0.764	0.784	0.609	3.942	0.434	0.035	58.214
	Summer	H	76	50	1.316	1	1	0.5	1	0.961	0.018	58
	Autumn	C	146	196	2.685	0.792	0.801	0.311	1.971	0.877	0.019	50.51
	Autumn	T	162	290	3.58	0.819	0.828	0.796	2.957	0.854	0.022	53.793
	Autumn	H	129	261	4.047	1	1	0.061	1	0.92	0.032	52.49
	Winter	C	146	170	2.329	0.876	0.882	0.505	2.957	0.937	0.016	74.706
	Winter	T	153	283	3.699	0.828	0.801	0.63	3.942	0.843	0.024	71.025
	Winter	H	147	181	2.463	0.758	0.853	0.498	1.971	0.927	0.017	54.144

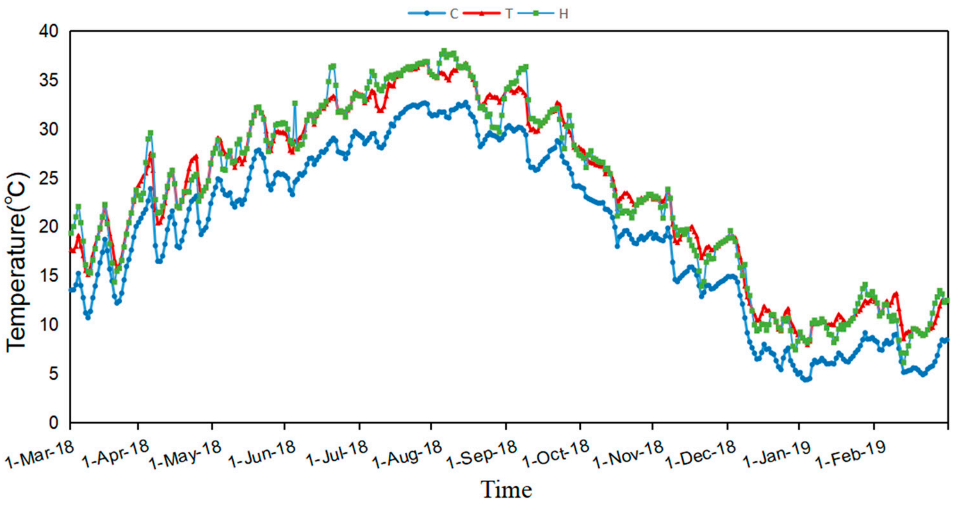

Figure S1. Temperature trend for all the treatments during the experiment. Each point on the curve represents the average temperature of the day. C, environment temperature; T, constant warming; H, heatwave.

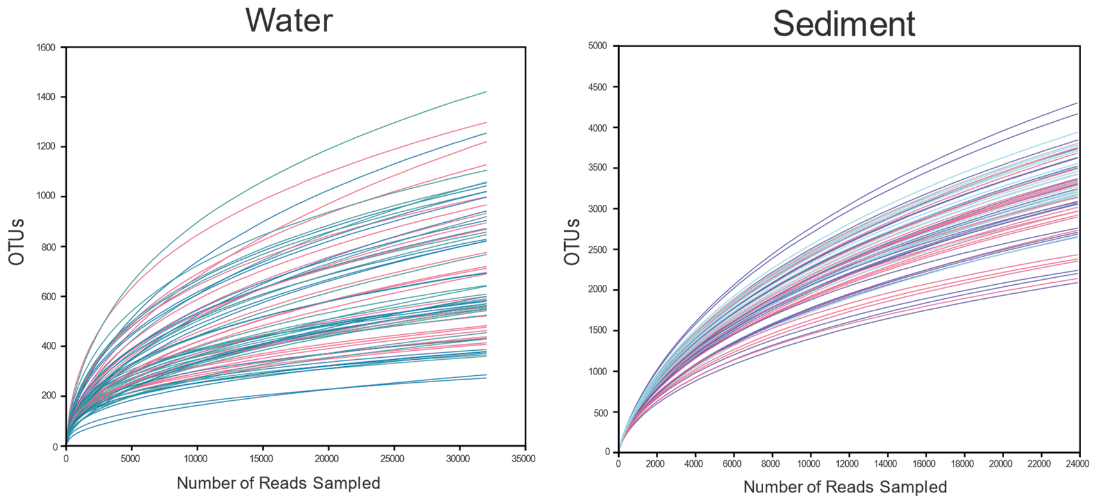

Figure S2. Rarefaction curves of water and sediments were constructed based on the number of reads sampled and the number of OTUS.

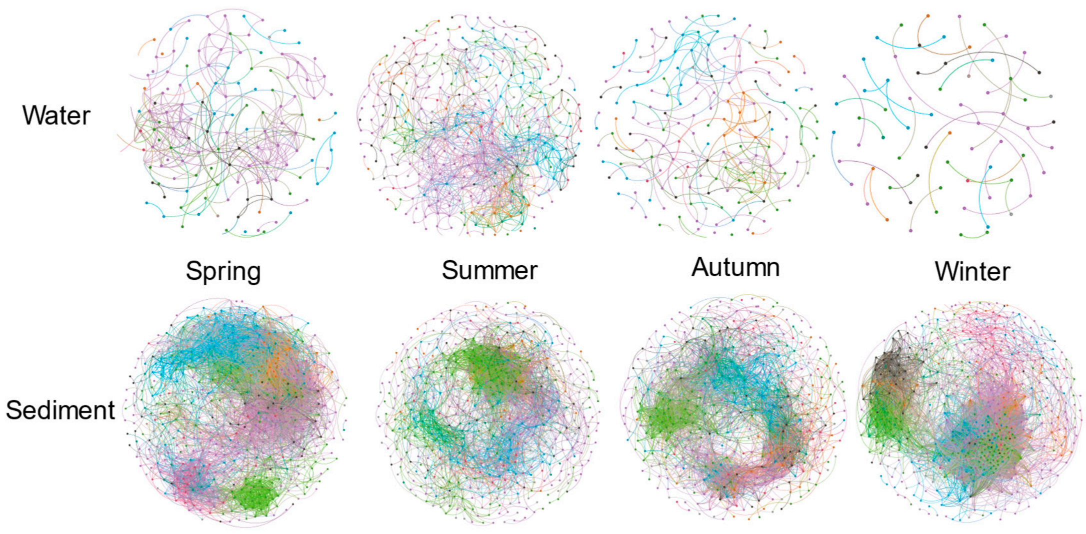

Figure S3. Co-occurrence network of bacterioplankton and sedimentary bacteria of four seasons. (The nodes represent OTUs with a total abundance greater than 0.1% in samples. Spearman correlation $\geq |0.6|$; $p < 0.05$). Nodes colors correspond to the phylum of bacteria community.
